# Lumbar Spine Ganglion Cyst: A Case Report

**DOI:** 10.7759/cureus.54934

**Published:** 2024-02-26

**Authors:** Fahad Alhelal, Sami AlEissa, Majed Abaalkhail, Raed M Almousa, Dana W AlDughiman, Hassan Khdary, Mohammed Alharbi

**Affiliations:** 1 Orthopedics, King Abdulaziz Medical City, Ministry of National Guard-Health Affairs, Riyadh, SAU; 2 Spine Surgery, Prince Sultan Military Medical City, Riyadh, SAU; 3 Medicine, King Saud Bin Abdulaziz University for Health Sciences, Riyadh, SAU; 4 Pathology and Laboratory Medicine, King Abdulaziz Medical City, Ministry of National Guard-Health Affairs, Riyadh, SAU

**Keywords:** lumbar spine surgery, lumbosacral radiculopathy, sciatica, spinal ganglion cyst, spine injuries

## Abstract

Ganglion cysts are typically periarticular soft tissue lesions commonly found in the wrist and forearm, with spinal involvement being rare. We present a clinical case of a 54-year-old female with a ganglion cyst at the L3-L4 level, causing radiculopathy symptoms. Despite initial difficulty in diagnosis due to MRI findings, surgical resection confirmed the extradural mass as a ganglion cyst. Postoperative recovery was uneventful, with immediate relief of radiculopathy symptoms. Challenges included distinguishing between synovial and ganglion cysts and accurately locating the cyst intraoperatively. This case highlights the importance of considering ganglion cysts in the differential diagnosis of spinal lesions and underscores the efficacy of surgical management for symptomatic relief.

## Introduction

Ganglion cysts are soft tissue lesions that develop in the periarticular tissue. While most commonly seen in the wrist and forearm, cysts in or around the spinal canal are rare and can cause radiculopathy symptoms [1]. According to literature, women between the ages of 20 and 50 are three times more likely to develop a ganglion cyst than men [2]. Spinal ganglion cysts (SGC) were frequently reported at L4-L5 due to the mobility of this spine level [3]. Regarding diagnosis and maximal treatment, MRI and surgical resection were the modalities of choice for both diagnosing and treating lumbar SGC [4,5]. Herein, we present a clinical case of a patient with an L3-L4 intraspinal cystic mass with an unclear etiology as reported by the MRI of the lumbar spine. Subsequently, the patient underwent decompression fusion removal of the extra/intradural lesion as the preoperative diagnosis was unclear. Surgical pathology confirmed the extradural mass was a ganglion cyst.

## Case presentation

A 54-year-old female, diabetic, hypertensive, and dyslipidemic, presented at our spine clinic with lower back pain associated with numbness and right radiculopathy for over a year. The patient had a history of using a cane while walking for assistance and was having difficulty sitting comfortably.

Clinical findings

**Figure 1:**
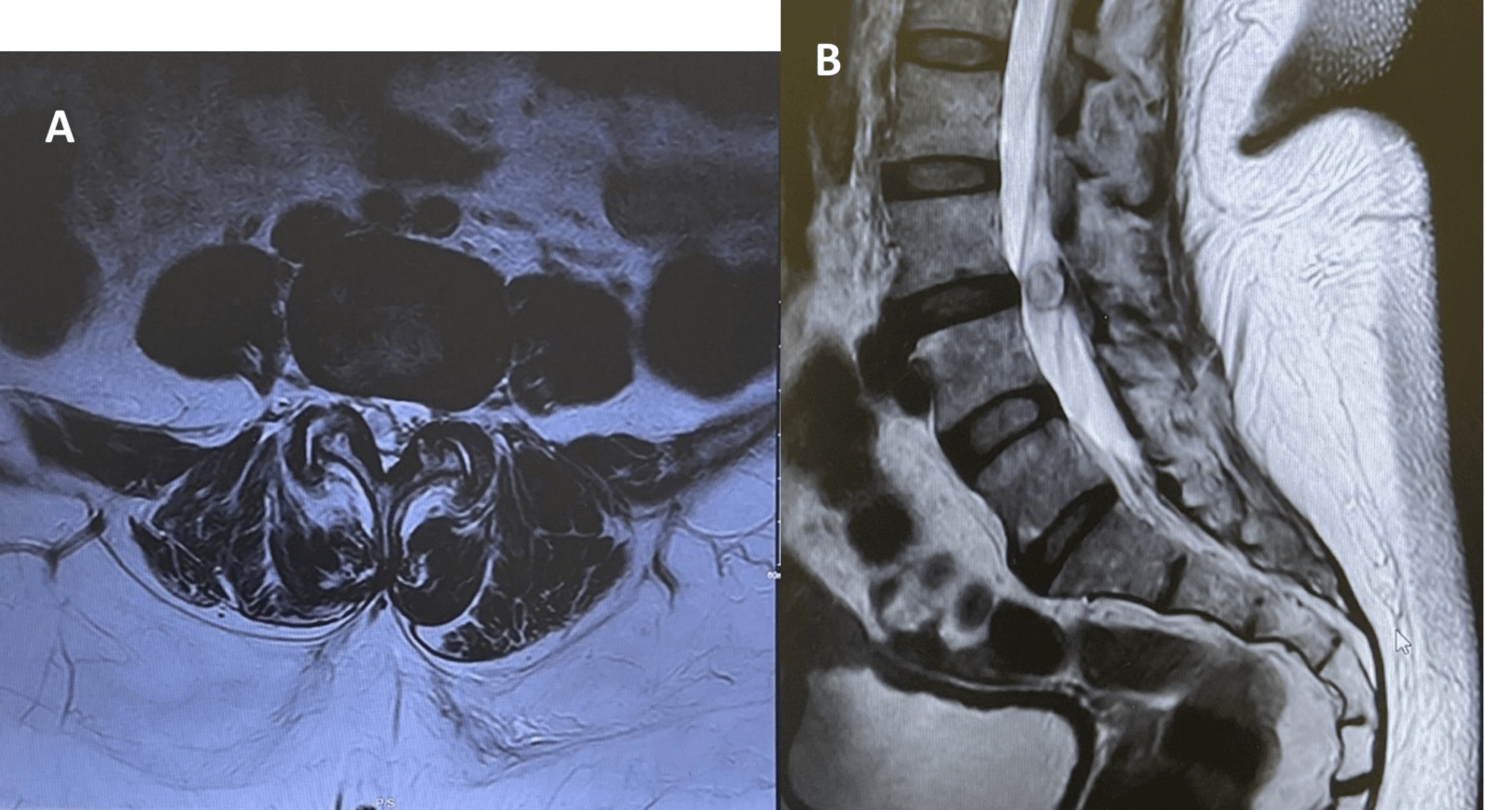
The patient's MRI findings Axial view (A) and sagittal view (B) show intraspinal lesions at L3–L4 with no disc bulging or herniation.

An examination was performed at the time of the initial presentation. It confirmed sciatica upon a positive straight leg raise test at 40 degrees of hip flexion. There was no neurological deficit. A full lumbar spine MRI confirmed the presence of a right intra/extradural rounded mass at the L3-L4 level, which was initially difficult to differentiate regarding the site with a benign-looking appearance that may represent a synovial cyst or facet cyst (Figures 1A, 1B). 

Therapeutic intervention

**Figure 2:**
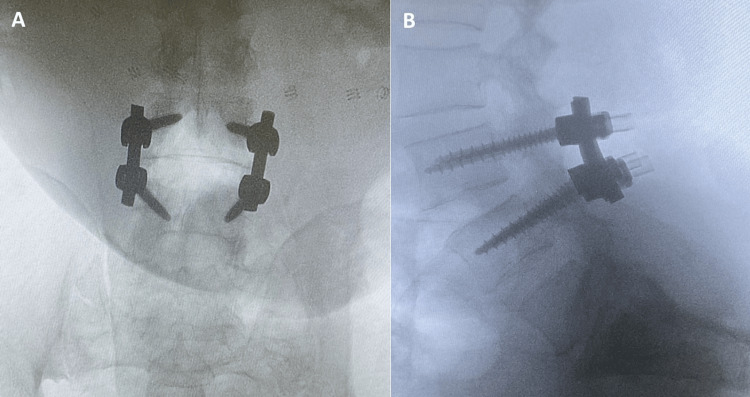
Intraoperative lumbar X-ray Anteroposterior (AP) view (A) and lateral view (B) post posterior decompression with rod and transpedicular screws at L3-L4 level. There were no hardware-related complications. The vertebral body heights and alignment are maintained. Post-surgical changes are noted.

After MRI confirmation, the patient was booked for decompression fusion of the L3-L4 intraspinal lesion. The procedure consisted of decompression with laminectomy bilaterally, and facetectomy was done only on the right side, preserving the left facet to enhance the fusion surface with a partial foraminotomy of it and a full foraminotomy on the right side. After removing the ligamentum flavum, we found inflammatory tissue surrounding the right L4 nerve root and extending to the midline of the dura. Under the use of an intraoperative microscope, using a rhoton, we did dissection all over the lesion, proximally, distally, medially, and laterally. The lesion was extradural, and we were able to remove it. We inserted a rod and transpedicular screws and did a reduction for the spondylolisthesis between L3 and L4. The X-ray showed good hardware position, and no post-surgical changes were noted (Figures 2A, 2B).

Histological description of the resected ganglion cyst

**Figure 3:**
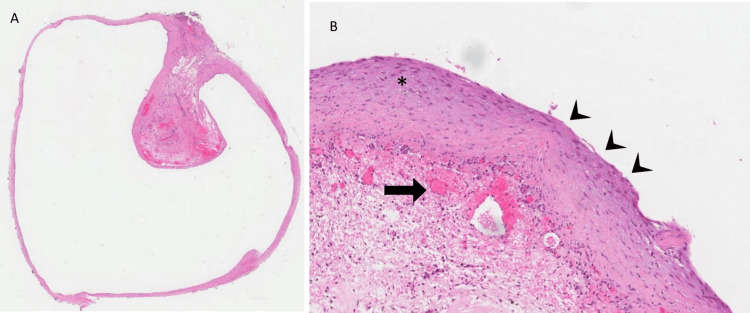
Hematoxylin & eosin stain The panoramic view of the cyst (A) shows that the cyst is unilocular. The 10x magnification of the cyst (B) shows that the cyst wall is composed of dense collagen with myxoid changes (asterisk) and no epithelial lining (arrowhead) and focal inflammation and granulation tissue formation (arrow) is seen.

Macroscopically, it consisted of an intact, unilocular gray soft cyst measuring 0.8 x 0.6 x 0.4 cm with a smooth and glistening outer surface. The cyst was filled with a gelatinous material with no papillary or solid components. The cyst's inner surface was gray and smooth, with a wall thickness of up to 0.1 cm. Microscopically, the cyst had a dense collagenous wall with focal chronic inflammatory cells (lymphocytes and plasma cells), focal granulation tissue and myxoid changes, and no true epithelial lining (Figures 3A, 3B).

Postoperative follow-up

The patient reported immediate relief of right radiculopathy postoperatively and was kept for five days in the hospital, where we started physiotherapy for mobilization purposes with spine precautions. The patient was discharged in good health and for outpatient clinic follow-up.

**Figure 4:**
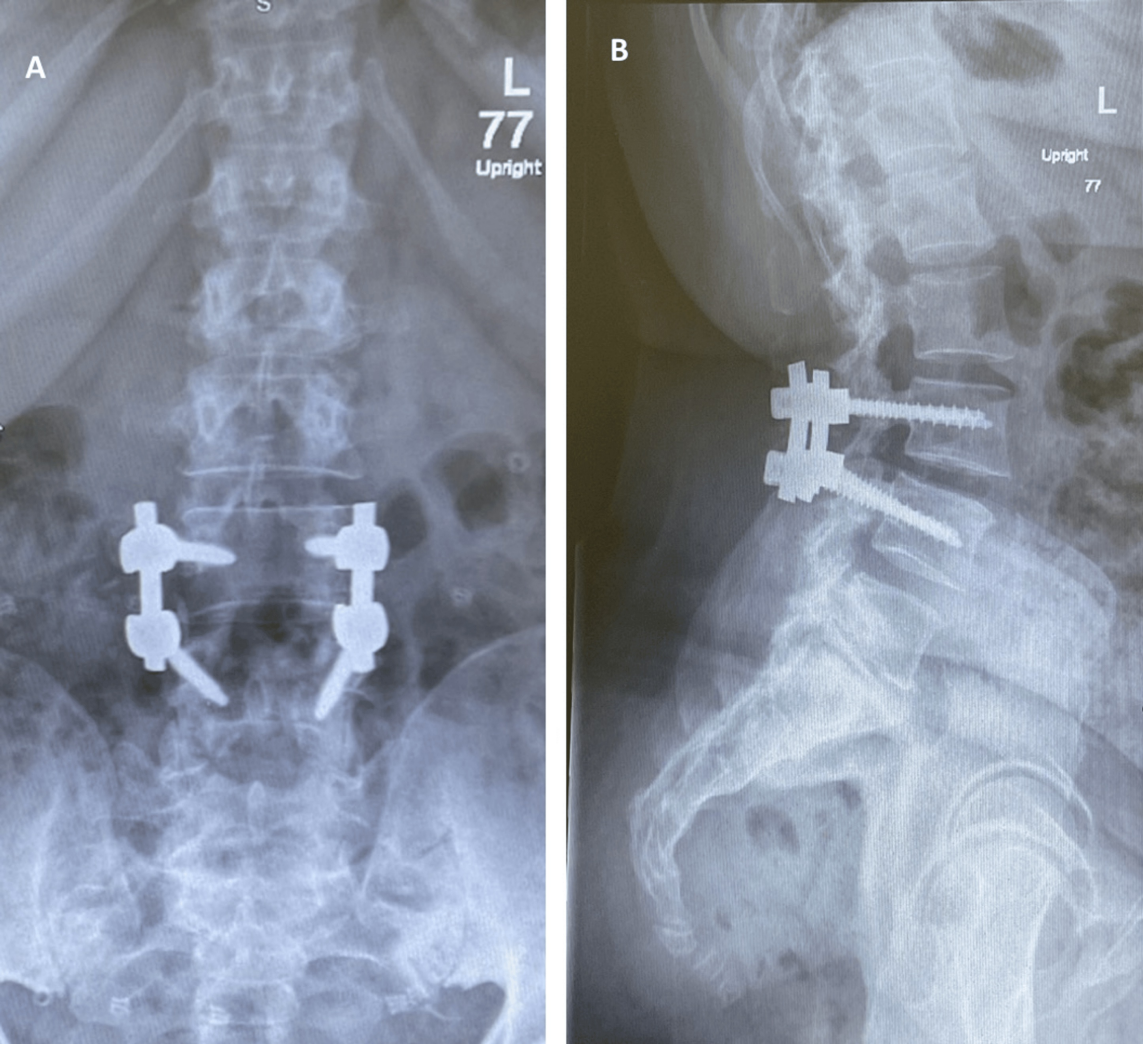
Postoperative lumbar X-ray Anteroposterior (AP) view (A) and lateral view (B) show no significant changes since the previous study.

Upon the three-week follow-up, the patient was doing fine with no active complaint, and the wound looked well with no signs of infection. The X-ray was unremarkable for any complications, and the reduction was successful (Figures 4A, 4B).

## Discussion

Ganglion cysts account for approximately 70% of cystic masses that are found in the hand and wrist [2], yet only a few data points show their presence in the spine. According to the literature, the etiology of a ganglion cyst remains unclear, suggesting that it could be related to facet joint hypermobility. This induced an inflammatory phenomenon that resulted in modifications of the articular synovial membrane, which led to cyst formation [6].

In our case report, the MRI showed that the cyst was a synovial cyst and not a ganglion cyst, and this is due to various factors. First is the nature of the location, which is rarely reported as a lumbar ganglion cyst. Second, the two terms are frequently used interchangeably in the medical field, with no clear criteria to distinguish between them. The differentiation between synovial and ganglion cysts is only of histological value, as they have similar clinical presentation and radiological characteristics. Histologically, synovial cysts are synovial cell linings filled with synovial fluid ranging from normal to inflammatory fluid [7]. On the other hand, ganglion cysts are delineated by dense fibrous connective tissue filled with a viscous content of hyaluronic acid and mucopolysaccharides [7]. In lumbar cysts, data suggest that both synovial and ganglion cysts originate from an extrusion of the synovium through a capsular defect from an unstable joint [8]. In terms of surgical management, instrumentation is required in a synovial cyst due to the instability of the joint, while in ganglion cysts, simple excision could be successful with minimal access [9].

The diagnosis of ganglion cysts depends heavily on history, physical examination, and MRI, with a sensitivity rate of 90% in comparison to CT scans [10]. Nevertheless, an MRI can have some limitations when differentiating between synovial and ganglion cysts, as presented in this case. Treatment of ganglion cysts is usually by surgical excision, as demonstrated in our management of this patient. What makes this case presentation impressive is the intraspinal/extradural location of the cyst, which gave it a distinctive presentation, along with the unclear nature and etiology of the cysts intraoperatively. In the literature, available data show that intraspinal/extradural cysts presented with sciatica-type back pain and leg pain [10]. These data support our patient's case presentation, giving ganglion cysts the importance of being implied in the future as one of the deferential diagnoses of sciatica pain symptoms. 

To our knowledge, only a few case reports have presented lumbar spine ganglion cyst management. With that being said, we have faced many challenges in managing this patient. First, the MRI findings were not compatible with the intraoperative findings. Second, the cyst was located in a complex location. Finally, the inaccuracy of the MRI to confirm whether it was an intradural or extradural cyst led us to identify it intraoperatively under the microscope.

## Conclusions

We presented a clinical case of a patient with an L3-L4 intraspinal cystic mass with an unclear etiology. Thereafter, the patient underwent decompression fusion removal of the extra/intradural lesion. Surgical pathology confirmed the extradural mass was a ganglion cyst. Our case report recommends including ganglion cysts as one of the differential diagnoses of sciatica. Also, further investigations and classifications should be conducted to differentiate between synovial and ganglion cysts, due to the unreliability of MRI in distinguishing between these cysts.
